# C_60_-Fullerenes: detection of intracellular photoluminescence and lack of cytotoxic effects

**DOI:** 10.1186/1477-3155-4-14

**Published:** 2006-12-14

**Authors:** Nicole Levi, Roy R Hantgan, Mark O Lively, David L Carroll, Gaddamanugu L Prasad

**Affiliations:** 1 Center for Nanotechnology and Molecular Materials and Department of Physics, Wake Forest University, Winston-Salem, NC 27105, USA; 2 Virginia Tech and Wake Forest University School of Biomedical Engineering and Sciences, Winston-Salem, NC 27105, USA; 3 Department of Biochemistry, Wake Forest University Health Sciences, Winston-Salem, NC 27157, USA; 4 Department of General Surgery, Wake Forest University Health Sciences, Winston-Salem, NC 27157, USA

## Abstract

We have developed a new method of application of C_60 _to cultured cells that does not require water-solubilization techniques. Normal and malignant cells take-up C_60 _and the inherent photoluminescence of C_60 _is detected within multiple cell lines. Treatment of cells with up to 200 μg/ml (200 ppm) of C_60 _does not alter morphology, cytoskeletal organization, cell cycle dynamics nor does it inhibit cell proliferation. Our work shows that pristine C_60 _is non-toxic to the cells, and suggests that fullerene-based nanocarriers may be used for biomedical applications.

## Background

Recent advances in materials science have fueled tremendous interest in numerous potential biomedical applications of various nanomaterials. For example, fullerene C_60 _molecules are unique for their multi-functional uses in materials science and optics [1-4], and are considered for a variety of biological applications (reviewed in [5]), such as imaging probes [6], antioxidants [7-9] and drug carriers (taxol) [10]. Our laboratory is interested in exploring whether novel multifunctional nanoparticles can be designed for cancer therapy and diagnosis. Realization of such a goal requires a better understanding of the interactions between nanoparticles and cells and it is important to determine whether or not the particles by themselves impact cell growth and differentiation. We have chosen C_60 _for initial studies because the established chemistries afford us the flexibility to couple various biologically interesting and relevant molecules.

However, some undesirable properties of C_60 _present specific challenges. For example, due to its inherent hydrophobicity, C_60 _is poorly soluble and naturally forms large micron-sized clusters in aqueous media. Therefore, organic solvents are routinely used for solubilization of C_60 _[11] Consequently, cell biological studies with pristine C_60 _have been limited.

Whereas chemical conjugation of C_60 _to various water soluble molecules improves the overall aqueous compatibility, pristine C_60 _is routinely dissolved in toluene [12,13], tetrahydrofuran (THF) [14] or other organic solvents, and then exchanged into water by extracting the organic phase with water. The resultant preparation is often referred to 'water soluble C_60_' which is typically of light yellow color and is estimated to contain a few hundred micrograms of C_60_/ml [15]. It has been suggested that the aqueous C_60 _is toxic to cultured cells and the toxic effects are due to peroxidation of lipids in cell membranes [16-19]. Various groups have reported that C_60 _(prepared using different methods) is not toxic [20-24] and some have attributed the toxicity of C_60 _to the side chains present on the functionalized C_60 _[25]. Possible mechanisms that might contribute to the observed toxicity of nano C_60_, include the solvent effects like atmospheric exposure of solvents such as THF (according to the manufacturer). Additionally, acquisition of ionogenic groups upon C_60 _crystal formation in aqueous media via THF solvent exchange have been reported to contribute to the potential biological consequences [26]. In support of these possibilities, a recent study suggests that toxicity of THF-derived water soluble nano C_60 _is abolished by removing THF by γ-irradiation. [27].

The conflicting data on cytotoxic effects of C_60 _merits attention and requires a resolution if these materials are to become biologically useful. The following simple hypothesis may reconcile with the mutually contradictory data on the cytotoxic effects of pristine fullerenes. C_60 _undergoes modifications during the preparation of water soluble C_60_, and such changes are responsible for the cytotoxic effects. Whereas the precise nature of such modifications is unknown at present, the hypothesis can be tested and the effects of C_60 _can be unequivocally examined if C_60 _can be applied to cells in such a way that obviates the need of preparing water soluble C_60_.

Studies presented in this manuscript examine the key issue of observed cytotoxic effects of C_60 _in cultured normal and malignant breast epithelial cells. We have developed a new, yet simple, method to directly apply C_60 _to cultured cells by modifying an established cell biological technique used in anoikis studies [28,29].

Although several key properties of fullerenes, such as the characteristic photoluminescence (PL) of C_60 _are well characterized in solutions [30] and polymer complexes [31], few have examined such properties in cellular environment. Photoluminescence of crystalline C_60 _occurs due to coupling of the vibrational modes of the lattice with electronic transitions and the PL signature of fullerene crystals may be useful to track the presence of C_60_. Results presented in this work demonstrate that unmodified C_60 _crystals are taken up by cells and intracellular C_60 _retains its optical properties, as determined by measurements of PL. Significantly, our studies reveal that C_60 _prepared by a variety of methods up to 200 μg/ml is not toxic to a number of cell types.

## Results and discussion

To eliminate the use of toxic organic solvents for applying C_60 _to cells, we have adapted methods routinely used in cell culture studies involving polymer coating of tissue culture dishes following solvent evaporation [28,29,32]. Colloidal suspensions of C_60 _in methanol (0.2 mg/ml) were prepared by sonication as described in Materials and Methods and applied to tissue culture dishes as an uniform coating. The organic phase is allowed to evaporate in a tissue culture hood, which leaves behind a coating of C_60 _on the dish. Cells are plated on to these dishes of C_60_. The C_60 _plated using this technique requires minimal manipulation and does not contain harsh organic solvents in cell culture. We refer to this preparation of C_60 _as 'methanol C_60_.'

### 1) Properties of methanol C_60_

**Figure 1:**
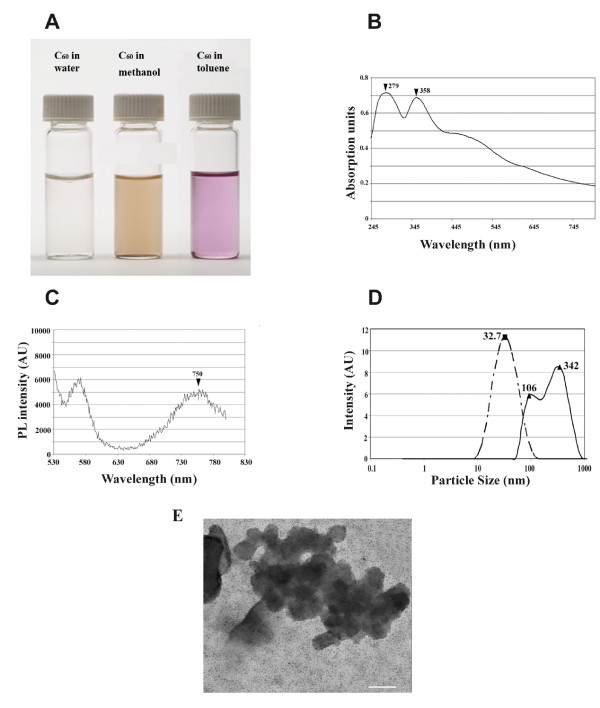
Physical properties of methanol C_60_. (A). Fullerenes suspended in water, methanol, and toluene. (B). UV/Vis absorption spectra of C_60 _suspended in methanol at a concentration of 0.2 mg/ml. (C). Samples were excited with 488 nm and PL spectra were recorded. (D). Measurements of particle size distributions of C_60 _in methanol (solid line) or in toluene (dashed line). (E) TEM micrograph of fullerene crystals in methanol drop-deposited onto a copper grid. Scale bar is 50 nm.

Sonication in methanol produces a uniform suspension of C_60_, which takes approximately 10–30 minutes to settle out of suspension. This slow rate of settling allows adequate time for recording of absorption spectra. Methanol C_60 _is a light brown colored suspension, indicative of large crystals in supension, compared to purple suspensions of toluene C_60 _which are known to contain significantly smaller sized crystals (Figure 1A). To characterize the physico-chemical properties of methanol C_60_, we determined its spectral features and measured the particle sizes of the colloidal suspensions of C_60 _in methanol. For example, C_60 _has a characteristic triplet-triplet absorption spectrum at 350 nm [33-35]. The absorption spectra of C_60 _in methanol was comparable with that prepared in toluene (λ_max _= 337 nm), which is more commonly used for suspending C_60 _(Figure 1B).

C_60 _exhibits a characteristic reddish orange PL signature in the solid state with a peak at 735 nm [31,36,37]. Methanol C_60 _retained this key property that is dependent on the interstitial spacing between C_60 _molecules in the crystalline structure with a broad peak around 750 nm (Figure 1C). These spectral findings are consistent with the established behavior of C_60_, which exhibits slight shifts in the absorption and PL peaks dependent upon the temperature [36] and the solvent used to disperse C_60 _[13]. Consistent with the properties described above, methanol C_60 _suspensions, when applied to tissue culture substrata, exhibited readily detectable crystal sizes and marked PL when visualized by light microscopy (discussed in the next section). Together, these data suggest that C_60 _remains adequately suspended in methanol and that the spectral characteristics are similar to those prepared in other organic solvents.

Particle size measurements confirm the stability of methanol- C_60 _suspensions. Dynamic laser light scattering measurements show that toluene C_60_, used as a reference (Figure 1D), yields uniformly sized particles with a mean size of 32.7 nm, consistent with published data [18,38]. Parallel measurements with methanol C_60 _reveals two peaks at 106 nm and 342 nm size, which indicates heterogeneity in the particle size (Figure 1D).

Transmission electron microscopy (TEM) was used to verify cluster sizes of fullerenes dried from methanol (Figure 1E). Methanol C_60 _clusters were observed in a wide range of sizes including large clusters in the micron range although many clusters smaller than 10 nm were observed. TEM micrographs corroborate particle size data obtained by dynamic light scattering which indicates the presence of a heterogenous mixture of variably sized clusters. Furthermore, following evaporation of methanol, the majority of fullerene clusters do not reaggregate, and have a range of sizes of tens of nanometers, although some larger clusters also exist. TEM data differ from that of the dynamic light scattering results in this regard since the light scattering apparatus accounts for the average of all sizes of fullerene clusters in solution.

Prolonged sonication of C_60 _in various organic solvents is routinely employed to prepare solutions of C_60 _[13,39]. As an additional measure to ascertain that suspension and sonication of C_60 _in methanol has not introduced any modifications into the fullerene, we analyzed each preparation by matrix-assisted laser desorption ionization time of flight (MALDI-TOF) mass spectrometry.

**Figure 2:**
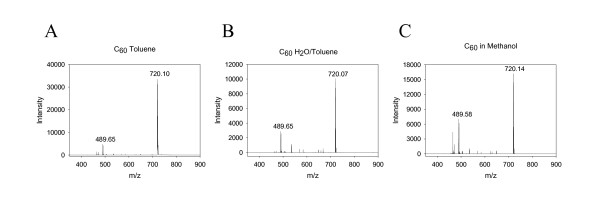
MALDI-TOF spectral analysis of C_60 _preparations. C_60 _was prepared in toluene (Panel A), in the water-soluble fullerene extracted from toluene (panel B) and in methanol (panel C). Representative aliquots of each preparation were analyzed by MALDI-TOF using α-cyano-4-hydroxycinnamic acid as the matrix. Spectra were acquired in the positive ion reflectron mode using the reflectron. The instrument was calibrated externally using a mixture of standard peptides (angiotensin II, 1046.54 Da; Substance P, 1347.736 Da; bombesin, 1619.823 Da; and ACTH clip 1–17, 2093.087 Da).

These analyses, performed in the positive ion mode, revealed a predominant species with a monoisotopic mass at 720.1 Da (theoretical mass of C_60 _= 720.00 Da) indicative of C_60 _preparations in methanol and toluene (Figure 2). The observed mass is consistent with the formation of a positively charged C_60 _ion by loss of an electron instead of gain of a proton. Interestingly, the same mass was observed upon analysis in the negative ion mode (data not shown). Each of the preparations contained a small amount of a species at 489.64 Da that was present in the original preparation of C_60_. In all cases, the principal component was pure C_60 _with mass 720.1 Da. The method of preparation in methanol or water used in this study does not appear to significantly alter the structure of the C_60_.

### 2) Growth of cells in presence of methanol C_60_

Previous studies have suggested that water soluble nano-C_60 _compromises the integrity plasma membrane, possibly due to lipid peroxidation [19]. To determine whether C_60 _applied to cells by a different method would produce a similar toxic effect, we have tested the effects of methanol C_60 _on cultured cells. First, we have examined whether C_60 _crystals are taken up by cells.

**Figure 3:**
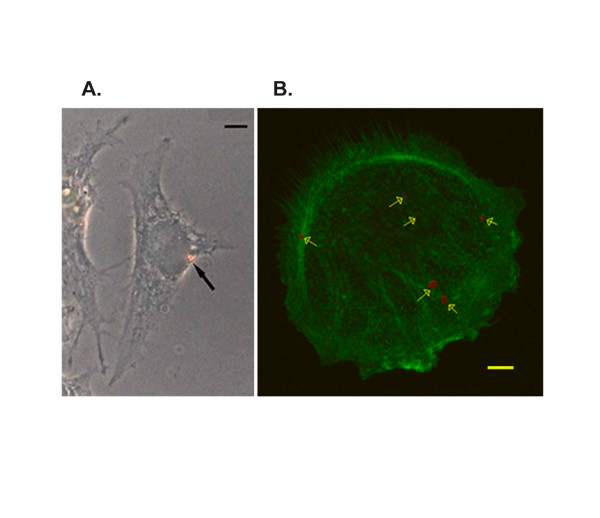
Cellular uptake of methanol C_60_. (A). Phase contrast image of a MDA MB231 cell which has internalized a C_60 _cluster. Intracellular C_60 _retains its PL signature. Scale bar is 20 μm. (B). Confocal microscopy of internalized C_60 _aggregates (red) identified with arrows. Methanol C_60_-treated MCF10A cells were plated on collagen coated chamber slides, fixed, counterstained with FITC-phalloidin. A compiled 3-dimensional projection of optically sectioned z-stack is shown. Scale bar is 5 μm.

Normal (MCF10A) and malignant (MDA MB 231 and MDA MB 435) breast epithelial cells were plated on either methanol-C_60 _coated dishes or control dishes and cellular morphology of the attached cells was examined. The presence of methanol C_60 _did not alter cell morphology or cell spreading and the PL signature of C_60 _is retained under normal conditions of cell culture. Further, we found that crystalline C_60 _is taken up by cells. To ensure that the nanoparticle is indeed internalized, the cells were trypsinized with trypsin to release them from the plate and replated on dishes coated with collagen I to enhance integrin-extracellular matrix interactions and cell spreading. Morphologically, cells cultured with methanol C_60 _re-attached and spread like the control cells. The fullerene nanocrystals retained their reddish orange PL, under phase contrast (Figure 3A) and bright field imaging used to ensure that the color of fullerenes is not due to an artifact of phase contrast.

The presence of intracellular C_60 _crystals was verified via examination through multiple focal planes using confocal microscopy. Normal breast epithelial cells (MCF10A) cultured overnight on methanol C_60 _were trypsinized, replated on collagen I, fixed in paraformaldehyde, extracted with 0.1% Triton X-100 and stained with FITC-labeled phalloidin for counterstaining. C_60 _crystals were readily evident by their characteristic reddish orange PL signature (Figure 3B). Multiple crystals of C_60 _of varying sizes were present in different focal planes, indicating their intracellular localization. Initial examination shows that intracellular C_60 _does not interfere with cell spreading on ECM or alter microfilament reorganization following attachment to ECM. Untreated (control) cells, processed in parallel, on the other hand, do not exhibit orange PL. Similar results were obtained with MDA MB 231 and MDA MB 435 breast cancer cells (data not shown). Since cytoskeletal reorganization following integrin activation involves a series of complex signaling events beginning with integrin activation and orchestrated activation of Rho GTPases [40], our results suggest that treatment of C_60 _is unlikely to interfere with the events following cell- ECM interactions.

### 3) Cell survival in presence of pristine C_60_

As discussed in the Introduction, there is a lack of consensus on the effects of C_60 _on cell growth, and we have hypothesized that the apparent cytotoxic effects of the nanoparticle are due to the methods of preparation and application of C_60 _to cells. Therefore, we have reassessed the effects of C_60 _on cell proliferation using methanol C_60 _and water soluble nano-C_60 _prepared from toluene.

**Figure 4:**
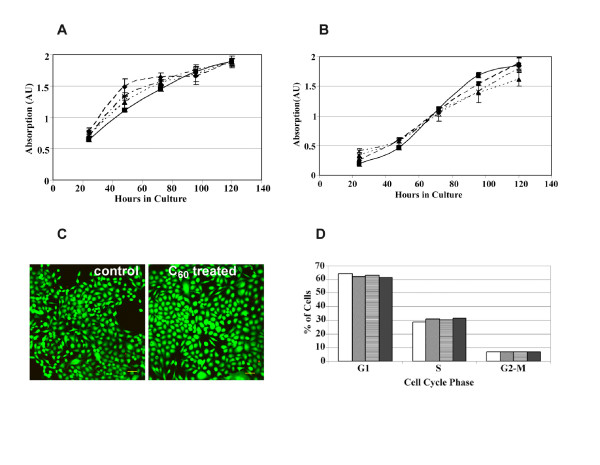
C_60 _does not inhibit cell proliferation. MCF 10A and (Panel A) MDA MB 231 (Panel B) cell lines were cultured either in the absence or presence of methanol C_60 _(0.2 mg/ml) and cell proliferation was assayed by crystal violet staining. ◆ Control, no C_60_, ■ 10 μg C_60_, ▲ 50 μg C_60_, **X **250 μg C_60_. (Panel C). MDA MB 231 cells were simultaneously stained with calcein and ethidium using a live-dead assay kit. Lack of red-colored cells and the presence of cells stained in green indicate the lack of toxicity (Panel D). MDA MB 231 cells were either untreated (open box □) cultured with varying amounts 10 (gray ), 50 (patterned ) and 100 μg (filled ■) of C_60 _for 48 h and analyzed for cell cycle progression by flow cytometry.

Several normal and malignant breast cancer cells were plated on tissue culture dishes pre-coated with various amounts (ranging from 10–200 μg (10–200 ppm) which corresponds to 13 nmoles to 277 nmoles) of methanol C_60_. Contrary to the published results which state that C_60 _is toxic at 20 ppb [18], culturing cells with significantly higher (200 ppm) concentrations of C_60 _did not adversely impact cell proliferation (Figure 4). The growth and proliferation of MCF10A (Figure 4A), MDA MB 231 (Figure 4B) was not affected by the presence of C_60 _and no cytotoxic effects were observed. Similar results were obtained with MDA MB 435 and HepG2 cells (see Additional file 1). Lack of toxicity of C_60 _on MDA MB 231 cells was further confirmed by 'live-dead' cell assays (Molecular Probes) (Figure 4C). Further, cell cycle profiles of MDA MB 231 cells cultured with or without C_60 _were essentially identical, indicating that the overall cell cycle parameters were unaltered (Figure 4D), and no subG_o_-G_1 _fractions (indicative of apoptotic populations) were evident in cells treated with C_60 _(not shown).

**Figure 5:**
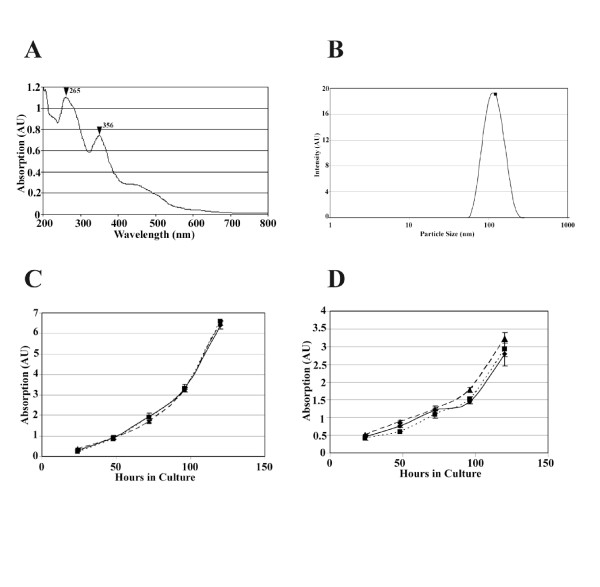
Water soluble toluene nano C_60 _also does not block cell proliferation. Absorption spectra (A) and particle sizes (B) of water soluble nano C_60 _from toluene are consistent with those reported in literature. The peak absorption wavelengths are indicated by arrows in A and the average particle size of the water soluble C_60 _is 122 nm. MDA MB 231 (C) and HepG2 (D) cells were cultured with 2.7 μg (dotted line) or 27.4 μg (dashed line) of water soluble toluene nano C_60 _or were untreated (solid line) and cell proliferation was assayed by crystal violet staining method.

Our finding that culturing cells with methanol C_60 _does not inhibit cell proliferation is at variance with published results [16,18,19,41], and hence we investigated whether the different methods of preparation and application of C_60 _would explain the differences in the effects of C_60_. We have prepared water soluble nano-C_60 _from toluene, using the published protocols [12,13] and characterized the material. Nano C_60 _prepared from toluene yielded 274 μg/ml (274 ppm) of lightly yellow colored water-soluble C_60_. Absorption spectra (Figure 5A) of nano C_60 _are in agreement with established spectral properties of C_60 _[33,35]. The particle size measurements of nano C_60 _revealed the presence of crystals with an average size of 122 nm (Figure 5B).

Culturing of MCF10A and HepG2 cells with up to 27.4 μg/ml (27.4 ppm) of water soluble nano C_60 _derived from toluene had no effect on cell proliferation (Figures 5C & D). The lack of cytotoxic effects was confirmed by two different assays (crystal violet staining and live-dead cell assays) and cell cycle analyses. The amounts of C_60 _used in these experiments is comparable to those used in previous studies where extreme toxicity was reported with other water soluble nano C_60 _preparations [18,19]. Thus, our findings with methanol C_60 _and water soluble nano C_60 _prepared from toluene demonstrate that cell proliferation is not inhibited by fullerenes and the nanoparticle does not exert toxic effects in cell culture.

Our efforts to increase the concentration of the nano C_60 _in cell culture studies is limited by the maximum concentration of C_60 _achievable in the water soluble preparation derived from toluene. Cell culture and proliferation in presence of other carbon nanomaterials, such as nanotubes, has also been successfully reported [42,43] and such findings are consistent with our data that show cell growth in presence of pristine C_60 _is feasible. While several researchers (for example, see [44,45]) report that nanotubes indeed are cytotoxic, a recent publication [46] attributes such toxicity to, at least, in part to technical issues. This is analogous to our hypothesis that methods of preparation of C_60 _accounts for the observed divergent cytotoxic effects of C_60_. Taken together, our data suggest that C_60 _particles can be utilized for the design and development of multi-functional nanoparticles and the core nanoparticle is unlikely to adversely affect cell physiology.

An important finding of this study is that C_60_, when applied as methanol suspension, is non-toxic to a variety of cell types and does not interfere with cell proliferation. This finding is supported by cell proliferation assays, cell cycle analyses and vital stains. Further, cells continuously cultured with C_60 _showed no defects in cell spreading and cytoskeletal organization, indicating the underlying cell-matrix interactions and signaling pathways are not adversely affected by C_60_. Our results are supported by other studies which show that C_60_, consistent with its well established electron acceptor properties, is a potent anti-oxidant [20,47]. This key finding differs from several published reports [16,18,19,41] which suggested that pristine nano C_60 _is toxic. To reconcile with the cell type differences, we have employed several normal and malignant epithelial cells and tested their proliferation in presence of toluene-derived water soluble nano C_60_. Some investigators have reported weak toxicity of a preparation of polyvinyl pyrrolidine (PVP) and C_60 _in cell culture and animal models compared to PVP alone [48,49]. However, it should be noted that the amount of C_60 _used in those studies significantly exceeded that used in the present work and the method of preparation of C_60 _is different.

Whereas several studies have examined the effects of C_60 _on a variety of cells, few studies have examined whether fullerene crystals are taken up by the cells. Confocal microscopy of methanol C_60_-treated cells onto collagen matrices reveals intracellular C_60 _nanocrystals of varying sizes in normal and malignant breast cancer cells (Figure 3B). We believe that this is a first demonstration of intracellular pristine C_60 _crystals using the PL signature as the reporter. The data shown in Figure 3B suggests that internalized C_60 _retains its crystal structure as evident from its bright reddish orange PL. While we demonstrate of larger C_60 _crystals in cells by confocal microscopy, smaller crystals (≤ 200 nm) may not be detectable by this technique. Recent reports indicate the ability to detect fluorescence of carbon nanotubes in cellular systems [50-54]. These findings suggest the possibility of detecting intracellular C_60 _fluorescence, although the signal is generally weaker than the infrared signal of nanotubes. While other nanoparticles such as functionalized nanotubes [55,56] and gold nanoparticles [57] are reported to be internalized through endosomal pathways, the route of internalization of pristine C_60 _is not known. Our data also suggest that the PL may be used as a reporting tool to estimate intracellular C_60 _levels, provided the yield from the smaller crystals can be quantitatively measured.

In summary, our work describes a simple and rapid method for application of C_60 _to cultured cells and to investigate the interactions of C_60 _with cells. We provide evidence that pristine C_60 _is taken up by normal and malignant cells and the intracellular C_60 _retains its PL signature. Finally, we demonstrate that continuous culture of cells with C_60 _is non-toxic and that cell adhesion, cytoskeletal reorganization following integrin activation and cell proliferation following treatment with C_60 _remain unaffected. The reported toxicity of pristine C_60 _is most likely due to incompletely understood solvent effects or to chemical modifications of the C_60 _that may occur during preparation. A key implication of our research is that fullerene-based nanoparticles could possibly be utilized for biomedical applications without negative consequences from the fullerenes themselves.

## Conclusion

C_60 _fullerenes are useful for several biological applications. Here we described a new and simple method of applying these materials to cells and shown that they are taken up by cells. Significantly, we demonstrate that unmodified C_60 _fullerenes are not toxic to cells. This finding should clarify the issue of perceived toxic effects of fullerenes and enhance developing novel biomedical applications using these nanoparticles.

## Materials and methods

### Fullerene suspensions

C_60 _fullerenes (Sigma Chemical Co) were sonicated in methanol at 0.2 mg/ml using a water bath sonicator (Branson) for 30 minutes to create a suspended fullerene solution which is referred to as methanol C_60_. 'Water- soluble' nano C_60 _suspensions were prepared from toluene using published procedures [12,13]. To prepare a 'nano-C_60_' suspension from toluene 0.5 mg of C_60 _was added per ml of toluene. The suspension was sonicated for 10 minutes in a water bath (Branson) until a uniform purple solution was obtained and all C_60 _had been dissolved as determined by observation. Following sonication in toluene, an equal volume of deionized water was added to the toluene/C_60 _suspension and an organic/water phase separation was observed. This solution was sonicated in a water bath until all the toluene had evaporated (no more purple solution left), typically requiring about 2–6 hours depending on batch quantity.

### Light spectroscopy

Fullerene suspensions were characterized by UV/Vis absorption (Beckman DU7500 spectrometer) and fluorescence spectroscopy. Photoluminescence (PL) measurements were made using a Safire_2 _multifunctional monochromator based microplate reader (Tecan Instruments). Because methanol C_60 _suspensions settle rapidly, spectra were recorded within 10 minutes of sonication.

### Particle sizing

Size measurements of the colloidal fullerene suspensions prepared from methanol and toluene were carried out using a light scattering Zetasizer Nano-S light scattering instrument (Malvern Instruments, Southboro, MA). Sonicated methanol C_60 _suspensions were immediately measured to prevent settling of the particles. Recording of the spectra was routinely completed within 10 minutes of sample sonication.

### Transmission electron microscopy

Transmission electron microscopy was done on fullerene clusters dried from methanol onto formvar grids. A Phillips TEM Transmission Electron Microscope (model 400, 120 keV) was used and a sample of C_60 _in methanol was dried onto a formvar grid for observation of the clusters.

### MALDI-TOF

An Esquire MALDI-TOF mass spectrometer (Bruker Daltonics Instruments, Billerica, MA) was used to measure the masses of molecular species present in the various C_60 _preparations. Solutions containing C_60 _were mixed with equal volumes of saturated matrix solution (10 mg α-cyano-4-hydroxycinnamic acid per mL of 0.05% trifluoroacetic acid and 25% CH_3_CN). Mass spectra were recorded in positive and negative ionization modes using the reflectron mode and calibrations were performed using a peptide mass calibration kit supplied by Bruker Daltonics.

### Cell lines

Normal (MCF10A) and malignant (MDA MB 435 and MDA MB 231) human mammary epithelial cell lines, and human liver carcinoma cell line (HepG2) were obtained from the American Type Culture Collection (Manassas, VA) and cultured under standard conditions.

### Cell culture

Methanol C_60 _suspensions were prepared and immediately applied to 12-well tissue culture dishes based on a protocol used for anoikis assays [29,32]. Following application of the suspensions, methanol was allowed to evaporate from the culture dishes while standing open in a sterile hood. Cells were plated onto the coated dishes and cultured in regular growth media in a tissue culture incubator. Cell proliferation was measured using crystal violet assays [58]. Culture dishes were rinsed with phosphate buffered saline (PBS) and stained in crystal violet stain (0.25% w/v in 50% methanol) for 10 minutes. Following rinsing of the dishes to remove excess stain, the dishes were air-dried, the protein-bound dye was solubilized in 50% methanol and the absorbance was recorded at 540 nm [59]. Each sample was measured in triplicate and the experiments were repeated at least twice. For some experiments, cell proliferation was assessed with a live-dead cell assay kit (Molecular Probes) containing calcein AM and ethidium dyes. Fluorescence microscopy was used to determine cell viablity by examining ratios of green (viable) to red (dead) cells.

### Flow cytometry

Cell cycle profiles were determined by flow cytometry using established protocols [29,60]. Cells were trypsinized and fixed in 70% ethanol for at least 24 h at 4°C, stained with propidium iodide and subjected to flow cytometric analysis on a BD FACStar instrument. The DNA content of cells in various phases of cell cycle was determined by Modfit program.

### Light and confocal microscopy

All cells lines were incubated with 200 μg of C_60 _from the methanol preparation for 24 hours at 37^0^C. Following incubation, cells were extensively washed with PBS to remove adherent extracellular fullerene clusters, trypsinized, and replated on collagen I coated (5 μg/cm^2^) chamber slides [32]. Samples were either directly viewed by phase contrast microscopy using an Olympus microscope or processed for confocal microscopy. Light microscopy images were recorded with a standard white light source without a UV filter. For confocal microscopy preparation, samples were fixed in 4% paraformaldehyde, extracted with 0.5% Triton X-100, incubated with FITC- labeled phalloidin (Molecular Probes) to visualize actin cytoskeletal filaments, and mounted with the anti-fade kit (Molecular Probes) [32,60]. Samples were viewed on a Zeiss LSM 510 confocal microscope. Detection of C_60 _was accomplished by excitation at 458 nm and the use of a long pass filter for λ > 650 nm. Images were optically sectioned and the projections of the compiled z-stack were imported into Adobe Photoshop (version CS2).

## Competing interests

The author(s) declare that they have no competing interests.

## Authors' contributions

NL, ML and GLP performed the experiments. RH and DLC helped in designing some experiments and interpretation of the data. GLP designed the overall project and wrote the manuscript, with inputs from other authors towards the final draft.

## Supplementary Material

Additional File 1 Effect of methanol C_60 _on the proliferation of cultured cells. MDA MB 435 breast carcinoma (A) and HepG2 liver carcinoma (B) cells were cultured under control or in the presence of methanol C60 (0.2 mg/ml) and cell proliferation was measured as described in the legend for Figure 4A and 4B. Click here for file
