# Supplementary figures and images for: C60-Fullerenes: detection of intracellular photoluminescence and lack of cytotoxic effects
Source: J Nanobiotechnology. 2006 Dec 14;4:14. doi: 10.1186/1477-3155-4-14 (PMC1764419; doi:10.1186/1477-3155-4-14)

**A**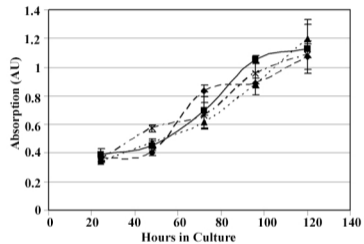**B**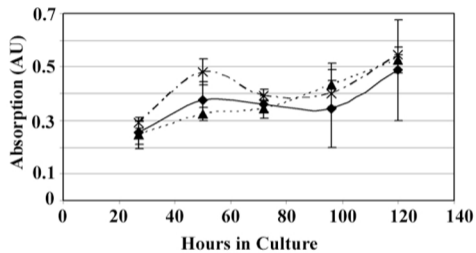

Supplement: Additional File 1 — Effect of methanol C60 on the proliferation of cultured cells. MDA MB 435 breast carcinoma (A) and HepG2 liver carcinoma (B) cells were cultured under control or in the presence of methanol C60 (0.2 mg/ml) and cell proliferation was measured as described in the legend for Figure 4A and 4B. [file 1477-3155-4-14-S1.pdf]
